# Standing Long Jump Performance in Youth with Visual Impairments: A Multidimensional Examination

**DOI:** 10.3390/ijerph18189742

**Published:** 2021-09-16

**Authors:** Adam Pennell, Nicole Yee, Carmen Conforti, Katienne Yau, Ali Brian

**Affiliations:** 1 Natural Science Division, Pepperdine University, Malibu, CA 90263, USA; nicole.yee@pepperdine.edu (N.Y.); carmen.conforti@pepperdine.edu (C.C.); katienne.yau@pepperdine.edu (K.Y.); 2 Department of Physical Education, University of South Carolina, Columbia, SC 29208, USA; abrian@sc.edu

**Keywords:** broad jump, horizontal jump, blind, skeletal muscle power, explosive strength

## Abstract

Muscular fitness, an important marker of health in youth, includes explosive strength, which can be assessed using the standing long jump (SLJ). Little is known concerning the SLJ in populations with disabilities such as youth with visual impairments (VI) who trend with decreased health- and performance-related outcomes. The purposes of this study were to investigate multidimensional SLJ performance outcomes in youth with VI (i.e., descriptives and percentages of occurrence) and to explore associations among such variables and known factors of interest (e.g., age) using robust linear bivariate regressions. This study was a secondary analysis from data collected in 2018 (*N* = 61, M_age_ = 12.98 years, SD = 2.21). SLJ performance was investigated using a multidimensional focus (e.g., distance, Test of Gross Motor Development-3 horizontal jump, landing developmental sequences, landing joint displacement, and stabilization after landing). In general, SLJ performance was substandard in youth with VI. Most SLJ assessment scores were predictive of other SLJ assessment scores. Few hypothesized variables of interest (e.g., multimorbidity) were predictive of SLJ performance. Youth with VI who match the characteristics of the current sample may have decreased explosive strength/muscular fitness and, worryingly, their SLJ performance may not be influenced by expected factors (e.g., age). Implications and explanations for these results are discussed.

## 1. Introduction

Muscular fitness is an important marker of health in youth [1] and is composed of various myo-specific dimensions such as muscular strength, explosive strength (colloquially referred to as muscular power), and muscular endurance [2]. There is strong evidence which supports an inverse relationship between muscular fitness and health risk factors such as adiposity, cardiovascular disease, and metabolic disorders [3,4]. Further, muscular fitness in youth likely has longitudinal effects, which may track into adulthood [3,4]. Thus, it is vital that muscular fitness is measured in youth in order to decrease or screen for potential health problems later in life [5].

As mentioned, one distinct component of muscular fitness is explosive strength (i.e., a functional characteristic), which is sometimes referred to as muscular power (i.e., a Newtonian metric). However, it is important to delineate such concepts. By physical definition, muscular power is determined by multiplying force by displacement and dividing the product by time (or by multiplying force by velocity). While commonly used to describe ‘fast speed’ muscular force in practical settings, muscular power is “generated and can be measured in any dynamic movement associated with an applied force, regardless of speed” [6] (p. 8). Thus, while attractive, the ability to produce ballistic, propulsive, high-velocity force-generating movements (e.g., jumping) should not be described as a measurement of muscular power unless the necessary Newtonian values have been computed (e.g., watts) [6]. Likewise, lower- and upper-body explosive strength field tests (which typically measure height or distance covered, e.g., seated medicine ball toss and vertical jump) are used as proxy measures of muscular power as performance on such tests is directly related to an individual’s achieved velocity, which is proportional to the amount of force generated during an action [2].

One field test that has garnered increased interest and relevance in recent years in youth populations has been the standing long jump (SLJ)—also known as the broad or horizontal jump. The SLJ is a lower-body explosive strength field-based assessment whereby an individual is instructed to jump as far as they can forward (i.e., horizontally in the sagittal plane) from a bipedal standing position with the functional goal of returning to and maintaining their original bipedal standing position. Historically, assessors have used distance covered during the SLJ as a surrogate measure of lower-body muscular power (i.e., greater jump distance is suggestive of increased muscular power), although assumptions and limitations concerning this relationship exist [2,6]. Importantly, the SLJ is a practical assessment that is inexpensive and easy to administer/interpret, while scores have been shown to have sound levels and forms of reliability and validity [7,8]. Thus, it is unsurprising that the SLJ has been recommended as a measure of musculoskeletal fitness by the National Academy of Medicine [2] and may be used as a general index of muscular fitness in youth [7]. 

Traditionally, the primary measurement of interest for the SLJ has been linear/horizontal distance jumped. Distance (e.g., meters and feet) is a quantitative (or product-oriented) outcome of SLJ performance. However, motor actions can also be assessed using a qualitative or process-oriented approach whereby movement patterns/technique are assessed [9]. While distance covered is a common (if not ubiquitous) SLJ heuristic, practitioners should consider using a combination of process- and product-oriented measurements (i.e., a hybrid orientation), as different classes of measurement provide shared—as well as unique—motor-related information or variance [9]. Thus, using both orientations of measurement would enable professionals to capture more holistic and salient descriptions or understandings of SLJ performance [9]. 

To this end, qualitative investigations into the SLJ (in all populations) have historically been monophasic in nature (i.e., increased focus on the takeoff phase of the SLJ vs. the flight or landing phases). However, with the recent publication of landing developmental sequences for the SLJ (i.e., a process-oriented assessment) [10], targeted SLJ landing studies are increasingly warranted—especially given that (while inter-connected) jump takeoff, flight, and landing phases have distinct intra-phase stages or events [11,12]. Ideally, the landing phase of a discrete SLJ is marked by decelerative eccentric contractions (i.e., joint displacement during landing), ground reaction force attenuation (i.e., a “soft/quiet” landing), and (subsequently) the ability to maintain one’s postural steadiness (i.e., static balance [referred to as stabilization by McKinley and Pedotti [12]). Thus, while the ability to produce lower-body explosive strength (e.g., jumping an increased distance) is important, it is reasonable to suggest that how an individual controls and/or translates that energy at landing may be equally as telling. Therefore, multiphasic SLJ investigations are needed and may lead to novel discoveries.

While the SLJ has been used to describe and track muscular performance within the youth population without disabilities [13], less is known concerning populations with disabilities such as children, adolescents, and youth (hereafter referred to as youth) with visual impairments (VI). In general, youth with VI have been found to be sedentary [14] and overweight [15]. Further, youth with VI have been shown to trend with decreased levels of health-related fitness, physical activity, and motor skill competence when compared to peers without VI [16,17]. Given these issues, it is possible that SLJ performance could be used as a valuable and utilitarian metric for identifying health-related discrepancies in youth with VI. However, an in-depth analysis of SLJ performance has never been completed in youth with VI. Further, due to limited investigation, uncertainties remain surrounding factors that may influence and/or be predictive of SLJ performance in youth with VI (e.g., age, height, degree of vision). Insights into such information would be valuable as discrepancies may be uncovered when compared to what has traditionally been found in youth without VI (e.g., being older in age and/or being a boy typically leads to increased SLJ distance [18,19,20]). Such findings would empirically inform what factors may (or may not) be influencing SLJ performance in youth in VI. 

No studies have completed an in-depth investigation of SLJ jumping and/or landing performance in youth with VI, constituting a significant gap in the knowledge base. Therefore, the purposes of this study were to (a) describe/compare SLJ performance to extant data in youth without VI using a hybrid measurement orientation and to (b) explore bivariate relationships between SLJ variables and potential variables of interest that may be predictive of SLJ performance (e.g., degree of vision) in youth with VI. It was hypothesized that, from a descriptive standpoint, SLJ performance would be diminished in youth with VI when compared to previously published data in youth without VI (e.g., norms). It was also hypothesized that SLJ assessment scores would be predictive of each other and that numerous variables of interest (e.g., age, sex, height, and vision level) would be predictive of SLJ performance in youth with VI.

## 2. Materials and Methods

### 2.1. Design and Setting

This study was a secondary data analysis [21]. Using a descriptive-analytic, cross-sectional design with convenience sampling, data for this study were collected at two Camp Abilities sites (Florida; New York) in 2018. Camp Abilities is a week-long overnight educational sports camp for youth with VI that follows a specific curriculum [22]. 

### 2.2. Participants

Sixty-one youth with VI completed the current study. Descriptive information for the sample were as follows: M_age_ = 12.98 years ± 2.21, M_height_ = 1.54 m ± 0.13, M_weight_ = 54.25 kg ± 20.97, M_BMI_ = 22.31 kg/m^2^ ± 5.79, M_BMI%ile_ = 69.22 ± 28.13, M_BMIz_ = 0.72 ± 1.03, M_maturityoffset_ = −0.22 years ± 1.77, M_lowerlimblength_ = 0.84 m ± 0.07, and M_seatedheight_ = 0.77 m ± 0.07. Forty-eight percent of the participants were girls (52% = boys), while 31% of the sample had a multimorbidity (i.e., dichotomous yes/no classification; defined as the coexistence of at least two chronic conditions [23]). 

Participants varied in degree of vision per the United States Association for Blind Athletes visual classification scale [24,25]. The United States Association for Blind Athletes scale consists of four levels (i.e., B1, B2, B3, B4) with B1 representing the lowest level of visual acuity (i.e., no light perception in either eye up to light perception, and an inability to recognize the shape of a hand at any distance or in any direction) to B4 representing the highest level of visual acuity (i.e., from visual acuity above 20/200 and up to visual acuity of 20/70 and a visual field larger than 20 degrees in the best eye with the best practical eye correction). Individuals can also be classified as B2 (i.e., from ability to recognize the shape of a hand up to visual acuity of 20/600 and/or a visual field of less than 5 degrees in the best eye with the best practical eye correction) or B3 (i.e., from visual acuity above 20/600 and up to visual acuity of 20/200 and/or a visual field of less than 20 degrees and more than 5 degrees in the best eye with the best practical eye correction). Concerning visual classification of the sample, seven were B4, 23 were B3, 11 were B2, and 20 were B1.

### 2.3. Variables and Instrumentation

#### 2.3.1. Qualitative (Process) Measures

*Test of Gross Motor Development-3 (TGMD-3) Horizontal Jump.* The TGMD-3 [26] is a process-oriented norm-referenced assessment used to evaluate motor skill competence in children aged 3 years to 10 years and 11 months and is composed of a locomotor subscale (e.g., run, horizontal jump, hop) and a ball skill subscale (e.g., throw, kick, and catch). Due to the developmental nature of the TGMD-3 [27], it is reasonable to suggest that if an individual older than 10 years 11 months does not achieve elevated or maximal scores on the TGMD (i.e., ceiling effects), raw scores can be used for analysis (i.e., criterion-reference) [28]. Brian and colleagues [29] previously provided modifications for administering the TGMD-3 to youth with VI. Further, TGMD-3 scores have been shown to have stout measurement properties in youth with VI (age range = three to 18 years) [29]. 

Concerning the TGMD-3, only the total, raw horizontal jump score from the locomotor subscale was utilized for the current study. The process-based scoring for the horizontal jump consisted of four criteria per trial (i.e., if a criterion was not observed during a trial, a score of ‘0′ was given; if a criterion was observed during a trial, a score of ‘1′ was given) summed across two scored trials. Therefore, raw scores for the TGMD-3 horizontal jump ranged from zero to eight points with high scores indicating better motor performance. 

*SLJ Landing Developmental Sequences.* Developmental sequences using a component (i.e., body part) approach for the SLJ landing were introduced by Lane et al. [10]. Specifically, this assessment was used to identify component-based actions of the shank, foot, and arm at landing during the two scored TGMD-3 trials. For each component, scores (i.e., levels) ranged from one to four (i.e., shank = 1–2 levels; foot = 1–3 levels; arm = 1–4 levels) with higher scores indicating developmentally advanced SLJ landing performance. Levels from each scored trial can be summed to give an ‘overall’ ordinal-like SLJ landing score (range = three to nine), can be analyzed at the individual component level (e.g., percentage of occurrence), or can be concatenated to provide a qualitative SLJ landing ‘profile’ (e.g., Level 1 shank, Level 2 foot, and Level 3 arm = 1-2-3 profile). Given the benefit of using a summed/cumulative score (i.e., higher scores = better performance), examining the prevalence of levels/scores within individual components (i.e., a gauge of *how* participants are landing the SLJ at the component level), and investigating categorical SLJ landing profiles (i.e., a multicomponent view of *how* participants are landing the SLJ), all three interpretations were adopted for use within the current study. 

To aid in interpretation of the individual component results, we extracted the estimated percentage of occurrence for the shank, foot, and arm components based on the graphical trajectories provided by Lane and others [10] from youth without VI (i.e., descriptive comparison group). Specifically, we extracted estimated percentages of occurrence using 13 years of age as the referent age as the average age of the sample was ≈13 years (M = 12.98). While such values were not directly comparable, it was believed that these descriptive comparisons would offer supplementary contextual information concerning SLJ landing developmental sequence performance in youth with VI. 

*Joint Displacement—Landing Error Scoring System (LESS*). The LESS is a 17-item clinical assessment which was developed to assist in the identification of potentially high-risk movement patterns (i.e., errors) during a depth jump-landing maneuver [30]. While the full LESS assessment is typically used in athletic/injury prevention-type investigations, one specific item from the LESS (i.e., item 16: joint displacement) was adopted for use within the current investigation. This metric was utilized as none of the previous assessments explicitly evaluated landing displacement style (e.g., stiff landing). Such an addition was important as an individual’s level of joint displacement at landing is indicative of factors such as eccentric deceleration/braking and force attenuation [31,32,33]. Thus, it was concluded that the displacement item from the LESS (which was concurrently scored with the TGMD-3 trials) would provide additional insight into the SLJ landing performance of youth with VI. Specifically, SLJ landing was coded as soft (0; large displacement of the trunk/hip/knee), average (1; some displacement of the trunk/hip/knee), or stiff (2; little [if any] displacement of the trunk/hip/knee) as viewed from the side/sagittal plane with lower scores indicating improved performance [30]. It is important to note that a soft landing may not always be advantageous depending on the goal/constraints of a task [34] and that a true, ‘ideal’ landing may not exist [35]. However, given that the current SLJ task was a discrete (i.e., a clear beginning and end point), ballistic action, it was concluded that a ‘soft’ landing was indeed suggestive of superior performance. 

*Supplemental SLJ Landing Postural Strategy/Intervention*. Finally, a novel item examining the use (or lack thereof) of a supplemental postural control strategy to maintain verticality at landing was investigated. Such a metric was believed to be useful for gleaning the level of stabilization at landing [12]. Specifically, participants received a score ranging from 0 to 3 depending on the sequence of events that occurred at/immediately following landing. Below are the criteria for each scoring option. It is important to note that, as previously mentioned, participants were not instructed or mandated to ‘stick the landing.’ Thus, each behavior was operationalized based on the authentic actions of the participants and the investigators’ previous experience with postural/motor skill assessment.

- None (0). Participants ‘stuck the landing’ meaning that after both feet contacted the ground, no additional steps, loses of balance, or falls occurred.
- Mild (1). Participants required ≈1 step to maintain their upright posture. Note that ≈1 step was used to allow for context. For example, a participant may have taken two steps, but the second step was seen as a natural and controlled ‘follow through’ step from the first step as individuals would sometimes take multiple steps after landing. The overall concept was to determine if individuals appeared to have full control of their upright posture within one step.
- Moderate (2). Participants clearly needed more than one step to help maintain their upright posture.
- Severe (3). Participants clearly stumbled or had a loss of balance/verticality whereby a significant intervention/effort was made and/or they fell to the ground.

#### 2.3.2. Quantitative (Product) Measures

*Distance*. During the official TGMD-3 trials, participants were instructed to stand at a takeoff line taped to the floor and were instructed to jump as far forward as possible. Linear distance covered in meters (m) was measured from the front of the toes at the takeoff line, to the point where the back of the heel nearest to the takeoff line landed on the floor [7]. As typically done in physical performance testing, the maximum value of the two trials was retained for analysis [7]. However, data from both trials will be presented. 

To aid with interpretation of maximal SLJ distance, age- and sex-specific percentile rank normative values were calculated for each participant using American [18] and European [19,20] SLJ norms. The American norms, while most recent from within the country, were somewhat antiquated. Caution should be exercised concerning such percentile ranks given that it has been suggested that high- and upper-middle-income countries may have been experiencing secular declines in SLJ performance in youth without VI since the year 2000 [36]. Likewise, the European norms were not directly translatable to the current American sample. However, it was believed that using such percentile ranks could provide additional contemporary/contextual information surrounding SLJ performance as it is not uncommon for European countries to use American-derived motor skill norms and values [37,38]. Percentile ranks are normally used to interpret individual performance; however, we opted to calculate the within-sample median percentile rank from the American and European percentile rank results. It was believed that this process would provide a broad and triangulated interpretation of SLJ distance performance within the current sample. 

#### 2.3.3. Potential Variables of Influence

Numerous variables may be predictive of (or influential on) SLJ performance/relationships in youth with VI. For example, certain variables may act as cofounders, covariates, mediators, or moderators [39,40] in relation to SLJ performance in youth with VI. While an exhaustive investigation was beyond the purposes of the current study, it was concluded that it would be beneficial to identify if certain extraneous variables of interest were predictive of SLJ performance. Outcomes from such analyses could (a) elucidate whether certain factors appear to currently influence (or not) SLJ performance in youth with VI (e.g., Currently, are there discrepancies in how certain extraneous variables associate with SLJ performance between youth with and without VI?) and/or (b) inform future SLJ investigations in youth with VI (e.g., Which traditional and/or novel variables of interest should, at the very least, be collected and/or considered when examining SLJ performance?). The variables of interest (and brief rationales for said variables) that were examined in the current study are listed below. It is important to note that, although inclusive, the investigated variables within the current study were not an exhaustive representation of all potentially-relevant extraneous variables which may influence SLJ performance in youth with VI (e.g., congenital versus acquired VI was not investigated).

*Demographics*. In general, both age and biological sex have been shown to influence SLJ jumping distance (i.e., increased jumping distance with increased age and in boys) [18,19,20]. Likewise, improvements in SLJ developmental sequences or components have been shown to be related to aging [10]. Maturation status has also been known to influence physical performance [41]. We examined estimated maturity offset (±), which has been defined as the estimated time (in years) before or after peak height velocity [42]. 

*Anthropometrics*. Weight, standing height, and leg length are biomechanical variables, which may influence and/or be predictive of SLJ performance (e.g., increased stature = improved performance [10,41,43]). Likewise, Body Mass Index z-score (BMIz), a function of weight and height that considers age and sex [44], was also investigated. 

*Additional Characteristics*. Specifically concerning youth with VI, degree of vision has been suggested as a potential determinant of motor skill performance (lower vision = lower performance [45]). Further, although traditionally overlooked in the realm of motor performance, Pennell [46] suggested that multimorbidity status (e.g., yes/no) should be increasingly investigated as a potential variable of influence in youth with VI (yes = diminished performance). 

### 2.4. Procedures

Following Institutional Review Board approval (ID: Pro00072384) and prior to data collection processes, all participants and their adults/guardians provided written informed consent. During face-to-face recruitment at each site (i.e., convenience sampling), a self-report demographic and visual information questionnaire was completed in tandem by each participant and their adult/guardian. 

Participant weight as well as standing and seated heights were assessed using a portable scale and stadiometer while barefoot, respectively. Further, while standing, participant right leg length was measured from the anterior superior iliac spine to the most distal portion of the medial malleolus [47] using a flexible, vinyl tape measure. Weight and standing height were used to determine BMI, which was subsequently converted to age- and sex-adjusted percentiles and z-scores (BMIz) [48] using *lamba* (L; power in the Box-Cox transformation to achieve normality for skewness), *mu* (M; median as the measure of central tendency), and *sigma* (S; dispersion as determined by the generalized coefficient of variation) parameters as specified by Cole and Green [44]. Likewise, sex-specific equations produced by Moore and colleagues [49] were used to estimate maturity offset. Participants were subsequently digitally recorded completing the TGMD-3 [26]. 

The TGMD-3 included one unscored practice trial prior to the two official/scored trials. Prior to the practice trial, all participants were provided with a visual demonstration of how to perform the horizontal jump. However, if it was clear that a participant required additional clarification on how to perform the horizontal jump immediately after their singular practice trial (as visual acuity could have be a limiting factor for the provided visual demonstration), appropriate, approved modeling techniques were utilized (e.g., tactile modeling) [29,50] to help ensure that horizontal jump performance was due to motor competence and not (for example) a lack of cognitive or visual understanding of the task. No visual demonstrations and/or modeling techniques were utilized following either of the official/scored trials. Modifications for administering the TGMD-3 to youth with VI were used pro re nata during the data collection process (e.g., one-person clapping in front of the participant to orient the jump direction) [29]. Further, as the TGMD-3 assesses naturalistic/authentic motor skill performance, participants were never given explicit instructions on a) how to jump/land, nor were they given b) knowledge of results or performance. However, participants were provided with the following verbal prompt before each horizontal jump: ‘Jump as far as you can!’.

For the purposes of this study, the two scored horizontal jumps from the TGMD-3 were qualitatively (e.g., SLJ landing developmental sequence) and quantitatively (i.e., SLJ distance) assessed using video analysis software (Dartfish, Fribourg, Switzerland). However, with the exception of the TGMD-3 horizonal jump score (which was a summed score of the two scored trials), the values that aligned with the trial with the largest SLJ were adopted for the primary analyses. For example, if a participant’s maximal SLJ distance occurred during their second trial, the following values from the second trial were retained for analysis: SLJ distance (m), landing developmental sequence score (3–9), LESS displacement score (0–2), supplemental SLJ landing postural strategy/intervention score (0–3). In contrast, the TGMD-3 score was determined by summing the criterion met across both trials (0–8) [26].

### 2.5. Data Anlaysis

Data were analyzed using R version 4.0.3 (R Core Team). Descriptive statistics (e.g., mean, median, measures of dispersion and shape) were calculated for both SLJ trials as well as the trial with the longest SLJ jump distance. Using the same trials, percentages of occurrence were calculated for (a) the SLJ landing developmental sequence results (i.e., component profiles and individual components [shank, foot, arm]), (b) the LESS joint displacement scores, and (c) the supplemental SLJ landing postural strategy scores. To aid with interpretation, within-sample median percentile ranks for the maximal SLJ distance values were calculated using previously published non-VI American [18] and European [19,20] percentile rank results. Likewise, extracted and estimated percentages of occurrence [10] using the rough average age of the sample as the referent age (i.e., 13 years of age) were used to descriptively compare the individual component SLJ landing developmental sequence results of the current sample to peers without a VI. 

Next, using scores that derived from the trial that had the longest SLJ distance for each participant (i.e., trial 1 or trial 2), robust linear bivariate regressions [51] were used to investigate for predictive relationships between the examined qualitative and quantitative SLJ metrics in youth with VI. Specifically, maximum SLJ distance (m), total TGMD-3 horizontal jumping score (0–8), the total landing developmental sequence score (3–9) were individually examined as response variables (i.e., *y*) with the respective SLJ assessment scores acting as singular explanatory variables (i.e., *x*). 

Likewise, potential variables of influence (e.g., age, sex, and multimorbidity) which could be predictive of SLJ performance were examined as singular explanatory variables (i.e., *x*) for all three of the previously described response variables/SLJ assessment scores (i.e., *y*) using robust linear bivariate regressions. Again, the SLJ scores (i.e., *y*) for these regression analyses was composed of values from the trial that had the longest SLJ distance. Analyses of the potential variables of interest enabled the current investigation to glean additional insights or discrepancies into the factors which may be (or are expectant to be) predictive of specific SLJ performance outcomes. 

For the robust linear bivariate regressions, evidence of statistical significance was supported if *p* ≤ 0.05. Further, robust adjusted *R*^2^ effect sizes (robust *R*^2^*adj*) [52] were calculated for each model and were interpreted as trivial (<0.02), weak (<0.13), moderate (<0.26), or substantial (≥0.26) [53]. 

#### Inter- and Intra-Rater Reliability

Several research assistants were trained by the lead researcher on how to quantitatively and/or qualitatively assess SLJ performance (e.g., software/coding training sessions, use of standardized coding documents). Further, intra- and inter-rater reliability of each variable of interest was assessed prior to all data analyses. Specifically, ≈25% (i.e., 16 participants) of each scored variable that was analyzed was randomly selected to (a) be re-coded by the same individual (intra-rater) and (b) be coded by a second, trained individual (inter-rater). All of the qualitative variables were ordinal in nature, therefore, AC_2_ agreement coefficients were calculated to determine inter- and intra-rater reliability (ordinal weighted) [54]. Likewise, for the quantitative metric (i.e., distance), intra-class correlation analyses using two-way mixed-effects, absolute agreement, single-rater models (3,1) were run per the recommendations of Koo and Li [55] to determine inter- and intra-rater reliability. Concerning the interpretation of the reliability coefficients, values of ≥0.90, ≥0.75, ≥0.50, and <0.50 were adopted as general guidelines indicative of excellent, good, moderate, and poor reliability, respectively [55,56]. 

## 3. Results

All reliability coefficients were greater than or equal to 0.90 suggesting excellent inter- and intra-rater reliability. With the exception of the TGMD-3 horizontal jump (M = 4.51, SD = 2.29, Mdn = 5, MAD = 1.48, Skewness = −0.52, Kurtosis = −0.68), it is important to remember that while there were two scored trials (which are presented), the trial with the largest SLJ distance (and the respective scores from that trial) were used for the main descriptive comparisons to extant literature (e.g., SLJ distance norms [18,19,20], individual component SLJ landing developmental sequence percentages of occurrence [10]) and for all robust linear regression analyses. General statistics for the SLJ assessments (other than the TGMD-3 horizontal jump) have been provided in Table 1. Overall, from a descriptive perspective, SLJ performance was suboptimal in youth with VI (e.g., diminished average maximal SLJ distance: 1.15 m [i.e., 3 feet, 9 9/32 inches or 3.77 feet]). The median percentile rank (of the entire sample) for maximal SLJ distance ranged from the 5th percentile using the historic, American norms [18] to the 10th [19] and 20th [20] percentiles using the contemporary, European norms. 

Table 2 provides the percentages of occurrence for several of the SLJ assessments. This categorical-inspired analysis highlighted that youth with VI had a proclivity to (a) land stiff, (b) require minimal-to-no supplemental postural control strategy immediately after landing, and (c) present with underdeveloped SLJ landing developmental sequence components/profiles. For example, the most common SLJ landing profile across trials 1, 2, and the maximal SLJ distance trial was the 1-2-2 profile (range = 32.79 to 37.70%; see Table 2). For reference, the highest SLJ landing profile was a 2-3-4 and only 3.28% (i.e., 2 participants) scored a 2-3-4 profile during trials 1, 2, and within the maximal SLJ distance trial. 

The prevalence of the 1-2-2 profile was echoed within the individual component results. It appeared that youth with VI may be more prone to landing with (a) an acute/perpendicular (versus obtuse) shank (Level 1), (b) a two-footed (i.e., simultaneous) and plantar-flexed or neutral ankle (i.e., forefoot or flat-footed) (Level 2), and (c) with a slight reach forward of the arms (Level 2). While speculative, youth with VI may be developmentally lagging behind sighted peers in relation to SLJ landing sequence performance based on the average age of the sample and estimated extracted values for a 13-year-old from Lane et al. [10] (see Table 2).

Individual robust linear bivariate regressions which investigated for predictive relationships between the selected qualitative and quantitative SLJ assessments can be found in Table 3. When attempting to predict the TGMD-3 horizontal jump, maximum SLJ distance, or total developmental sequence score, nearly all predictors were statistically significant. Exceptions were the supplemental postural strategy score (all *p*-values > 0.05, all robust *R*^2^*adj* = 0.00 [trivial effect sizes]) and the LESS joint displacement item when attempting to predict the TGMD-3 horizontal jump (*p* = 0.09, however, robust *R*^2^*adj* = 0.12 [weak effect size]). All other results were statistically significant with effect sizes ranging from weak to substantial (robust *R*^2^*adj* =0.12 to 0.42). 

Concerning the potential variables of interest across the three different response variables, being multimorbid was statistically significant when attempting to predict the TGMD-3 horizontal jump score (*p* = 0.006, robust *R*^2^*adj* = 0.09 [weak effect size]) and maximal SLJ distance (*p* = 0.001, robust *R*^2^*adj* = 0.12 [weak effect size]), while degree of vision was statistically significant when attempting to predict the total developmental sequence score (*p* = 0.03, robust *R*^2^*adj* = 0.03 [weak effect size]) (see Table 4). No other variables of interests were statistically significant.

## 4. Discussion

The purposes of this study were to (a) describe/compare SLJ performance to extant data in youth without VI using a hybrid measurement orientation and to (b) explore bivariate relationships between SLJ variables and potential variables of interest that may be predictive of SLJ performance (e.g., age) in youth with VI. First, SLJ performance in youth with VI will discussed followed by the regression results. 

The first hypothesis was confirmed as youth with VI (in general) presented with SLJ scores that were lower than what has been consistently published in youth without VI. On average, TGMD-3 horizontal jump performance was underdeveloped based on developmental expectations. The high score for the TGMD-3 horizontal jump was eight points, and based on the developmental nature of the assessment, participants have the potential to top-out (or be close to topping out) the assessment by 11 years of age [26]. As the average age of the sample was ≈13 years (M = 12.98), participants were (overall) only earning 56% or 63% of the possible eight points using mean- and median-based measures of central tendency, respectively (M = 4.51, SD = 2.29, Mdn = 5, MAD = 1.48). This finding was not surprising as youth with VI have been shown to have diminished TGMD-3 performance in both cross-sectional [29,57] and longitudinal investigations [58]. 

Likewise, the average maximal jump distance of 1.15 m was low relative to extant literature. After calculating the median percentile rank for the entire sample (i.e., group characteristic) using three separate normative arrays, SLJ jump distance performance was stark (i.e., 5th [historic American]; 10th or 20th [contemporary European] percentile ranks). Yet, since SLJ distance may have experienced a secular decline within the United States in recent decades [36], the median percentile rank for the current sample may be higher than the 5th percentile within the contemporary United States. Such a hypothesis may be explained by shifting percentile ranks. That is, modern American youth may not be jumping as far, and as such, present-day percentile ranks would ‘shift up’ meaning shorter jump distances today would achieve a higher percentile rank (from a historical perspective) [36]. Thus, a median 5th percentile rank may be accurate when considering past SLJ performance within the United States. However, it appears plausible that the true median percentile rank may range between the 10th and 20th percentiles as paralleled by the European data. Such conclusions will remain speculative until modern SLJ norms are calculated in American youth. Regardless, while we cannot designate percentile ranks to the current sample with sound and temporal accuracy, it is reasonable to suggest that maximal SLJ is likely poor in American youth with VI when compared to sighted peers.

The relatively low mean SLJ maximal distance should also be recognized as an *omen gravitas* by practitioners, researchers, and health professionals alike. Given that SLJ distance is a valuable marker of health/muscular fitness [1,3,4,7], concerns over the acute and longitudinal health/physical functioning of youth with VI should be raised. Such a conclusion is reinforced by the fact that muscular power performance has been shown to be a determinant of functional movement in, for example, older healthy and mobility-limited adults [59] and to be reduced in adults with VI [60,61]. Effectively, these findings suggest that youth with VI who match the characteristics found within the current sample may have decreased muscle mass, fast-twitch muscle fiber contractile properties, muscle quality, and/or neuromuscular function, which may have deleterious effects on various life domains (e.g., decreased activity, participation, body function/structure, see [62,63]). However, it is important to note that muscular power (and by extension explosive muscular strength as investigated herein) can be influenced by multiple physiological [64,65] and psychomotor factors (e.g., coordination, motor learning) [66,67]. 

Shifting back to the qualitative results, scores were not exceptionally high for the summed developmental sequence score for the SLJ landing relative to previously published data in youth without VI. Out of a minimal score of three and a maximal score of nine, youth with VI hovered around five total points (M = 5.31, SD = 1.36, Mdn = 5.00, MAD = 1.48). Currently, it is not known if such scores would differ from an age-matched sample of peers without VI. However, based on percentages of occurrence that were estimated/extracted from Lane and others [10] in youth without VI and by using 13 years of age as a reference (i.e., the rough average age of the current sample) (see Table 2), it is possible that youth with VI would generally present with a higher percentage of lower scores across all three body part components, and thus, might score (on average) lower than youth without VI. However, further investigation is needed as such a conclusion is speculative. 

Concerning the overall individual-component percentage of occurrence results, youth with VI were most prone to landing with an acute/perpendicular shank (Level 1), symmetrical plantar-flexed or neutral ankles (Level 2), and with a slight reach forward of the arms (Level 2). This was confirmed by the profile-based results, which showed that a 1-2-2 profile was, by and large, the most common landing profile across all trials (range = 32.79 to 37.70%). Concerning the other most common landing profiles, results were varied; however, it was interesting that only 3.28% (i.e., two participants) scored a 2-3-4 profile (i.e., the most advanced profile). It is important to note that based on cross-sectional data, increased levels of landing performance (especially concerning the foot and arm) appear to occur between the ages of 12 and 18 years [10]. As the average of the current sample was ≈13 years (range = 8.7 to 18.7 years), it was not expected that the majority of the sample would score a maximal developmental sequence landing score/profile. Yet, 25 participants, or 41% of the sample were 13 years of age or older. Thus, from a developmental perspective, it seems reasonable to suggest that an increased number of (for example) 2-3-4 profiles should have occurred based on the trajectories previously provided by Lane and colleagues [10] using youth without VI. In line with the previous lackluster TGMD-3 horizontal jump results, these results appear to suggest that advanced/elite-level landing performance (from a qualitative perspective) may be the exception as opposed to the rule in youth with VI. 

While profile-level data are informative, further inspection at the individual component-level was warranted. As previously mentioned, Levels 1, 2, and 2 were the most common scores for the shank, foot, and arm, respectively, in youth with VI. While results were mixed, only these levels and their implications will be discussed for brevity. Landing with an acute/perpendicular shank (Level 1) suggests reduced landing gain (i.e., decreased distance between the feet and the center of mass at contact) [10]. This finding is important as landing gain has been described as having excellent discriminatory properties across developmental stages [68] and may be predictive of SLJ distance [69]. Landing with a symmetrical neutral/plantarflexed foot action (Level 2) suggests a more advanced landing pattern (i.e., narrower base of support) when compared to the more rudimentary, one-footed/asymmetrical foot landing strategy (i.e., wider base of support). Based on this foot action result, it may be that a majority of youth with VI have developed some level of dynamic interlimb coordination and postural control in relation to the SLJ landing [10]. However, foot action concerns remained as only ≈5% of the sample landed symmetrically while dorsiflexed (Level 3 range = 4.92 to 6.56%) across all trials. It is probable that this foot action result is directly related to the previously described shank action result (i.e., high occurrence of acute/perpendicular shank at landing = decreased occurrence of foot dorsiflexion = decreased landing gain = decreased distance). Concerning the arm action, the most common occurrence was the slight forward reach (Level 2), which suggested that the majority of the sample were attempting to control their forward rotation (or angular momentum) rather than attempting to maximize their SLJ distance [10]. Using an arm strategy such as the slight forward reach could inhibit hip flexion, move an individual’s center of mass forward, and/or lead to a higher center of mass at landing—all of which may lead to inferior SLJ outcomes (e.g., shorter SLJ distance or landing gain) and/or be indicative of a less advanced landing strategy [10]. 

Concerning the LESS displacement item, it was overwhelmingly clear that youth with VI tended to land the SLJ stiffly (range = 72.13 to 75.41%). This result suggested that many youths with VI may not prepare for and/or execute a landing strategy marked by trunk/knee/hip joint displacement. Thus, youth with VI may not know how to or be comfortable with applying decelerative eccentric (i.e., braking) contractions across an increased range of motion and subsequently may also present with an inferior ability to absorb ground reaction forces at landing. This finding is important as landing with increased displacement (in general) is viewed as a protective strategy (i.e., produces decreased joint and musculoskeletal stress) [70]. It is possible that the increased prevalence of stiff landings may be partially due to the sample’s tendency to land with an acute/perpendicular shank, a two-footed forefoot or flat-footed, and/or a slight forward reach of the arms. 

However, an important question must also be raised concerning this result: Do youth with VI purposefully stymie their SLJ performance? Based on the low (average) SLJ maximal distance and the increased prevalence of stiff landings within the sample, it may be that youth with VI prioritize safety (e.g., stability; not falling at landing) as opposed to performance when asked to perform a maximum effort SLJ. This could be described as a stability-mobility trade-off whereby youth with VI adopt cautiousness in favor of efficient movement [71,72,73]. If this is indeed the case, youth with VI may be landing stiffly because they are not producing large amounts of explosive strength at takeoff and therefore, may not feel the need to employ a large amount of joint displacement at landing (i.e., less explosive takeoff = less need to land with increased trunk/hip/knee displacement). 

In contrast, lack of vision may negatively influence pre- and post-landing performance [11] as impaired vision may constrain an individual’s ability to produce an optimal level of spatial and/or temporal accuracy during the SLJ landing. This is especially relevant given that the SLJ is a skill that is generally marked by open-loop control (i.e., rapid, all-or-nothing movement; limited feedback during the movement [74]). Thus, youth with VI have two factors to contend with concerning the SLJ, (a) limited feedback during the attempt and (b) susceptibility to over- and/or under-estimating landing parameters (i.e., planning or feed-forward issue) due to a lack of or presence of conflicting visual information. Thus, stiff landing may be a common (or default) strategy in many youths with VI as such individuals may not be able to optimally reweight [75] or exploit available sensory information [76] and/or they may not be adept at judging or preparing for the SLJ landing. However, it has been suggested that vision level may play a minor (as opposed to major) role in landing performance and that landing performance may be influenced by a variety of elements such as reflexes, experience, and contextual factors [11].

Interestingly, a majority of participants either required minimal intervention immediately after landing (i.e., control within ≈1 step; range = 32.79 to 37.70%) or ‘stuck’ their SLJ landing (range = 37.70 to 44.26%). On the surface, the lack of postural intervention after landing could be viewed as a sign of advanced SLJ landing performance. However, it may be that this outcome was a byproduct of decreased explosive strength/SLJ performance (i.e., domino effect: hampered SLJ behavior/effort artificially inflated/improved supplemental postural strategy results). Again, this may be representative of the stability-mobility trade-off whereby many youths with VI may be self-selecting to produce limited lower-body explosive strength during the SLJ so that they do not have to negotiate with increased, stability-challenging Newtonian mechanics at landing (i.e., psychological preapprehension). However, an individual’s propensity to present with a stability-like profile (as delineated by the stability-mobility continuum) is likely due to a combination of constraints [77] such as psychological (e.g., preapprehension), psychomotor (e.g., motor development), and/or physiological (e.g., neuromuscular function) factors. Regardless, it was notable that ≈20–25% of the sample required a moderate (range = 13.11 to 19.67%) or severe (range = 4.92 to 6.56%) postural intervention following the SLJ landing, suggesting that—at minimum—a reasonable amount of youth with VI may struggle with achieving stabilization following the SLJ landing in naturalistic settings (i.e., lack of momentum control and force attenuation). As the current investigation did not necessitate a controlled landing, future research comparing landing technique, displacement, and supplemental postural strategies whereby youth with VI are instructed to stick or not stick the SLJ landing using quantitative (e.g., distance, kinematics, kinetics) and qualitative metrics are warranted (i.e., Would the current results hold across different task instructions?). Using Bernstein’s stages of learning as a guide, it appears that youth with VI, overall, are not highly coordinated standing long jumpers/landers as they do not appear to be efficient exploiters of passive dynamics (e.g., inertial, frictional, reactive forces [78]). 

Shifting to the robust linear bivariate regression results, it was not unsurprising that many of the SLJ assessment scores were predictive of each other as they all (hypothetically) assess similar and/or related components of SLJ performance (see Table 3). Thus, the hypothesis that the included SLJ scores would be predictive of each other in youth with VI was (in general) confirmed. Scores from the total/summed developmental sequence SLJ landing score (from the jump that had the largest SLJ distance), the TGMD-3 horizontal jump (summed from two trials), and the maximal SLJ distance (best of two trials) were consistently predictive of each other (robust *R*^2^*adj* ranges = 0.31 to 0.42 [substantial effect sizes]). While outside of the purposes of the current study, such results speak to the convergent validity (i.e., the extent to which two measures capture a common construct [79]) of these SLJ assessment scores in youth with VI. More saliently, these results suggest that (in general) there is value in using a hybrid, multiphase SLJ assessment orientation as each measurement appeared to provide shared as well as unique variance concerning the SLJ in youth with VI. While SLJ distance (e.g., maximal, average) is easy to administer, calculate, and interpret [7,8] and has been identified as a marker of health [1] and as a general index of muscular fitness in youth [7], the potential utility (e.g., predictive validity, status as a health or performance indicator) of the qualitative SLJ assessments used within the current study are unknown but should be investigated. Such scores may provide improved and/or novel information (compared to SLJ distance) concerning various health- or performance-related outcomes in youth with VI. 

However, there were mixed results between the various SLJ assessment scores. For example, the novel supplemental SLJ landing postural strategy/intervention item did not predict any of the three SLJ assessments (all *p* > 0.05, robust *R*^2^*adj* = 0.00 [trivial effect sizes]). It may be that the postural strategy/intervention item is measuring a characteristic that is independent of the included SLJ assessments, or a lack of variability/sensitivity/discrimination may be the culprit, as (for example) a limited number of participants scored a three (i.e., severe intervention = a significant effort was made, clearly stumbled or had a loss of balance/verticality, may have fallen to the ground). Yet, knowing that an individual has fallen (or may be prone to falling) during a SLJ attempt could inform an individualized SLJ intervention from a contextual standpoint. Thus, while the novel supplemental SLJ landing postural strategy/intervention item was not predictive of other SLJ assessment scores within the current sample, it stands to reason that the item could assist with providing a more holistic picture of SLJ performance or related (but less understood) SLJ outcomes in youth with VI (e.g., stabilization).

Likewise, the LESS joint displacement item was somewhat predictive of maximal SLJ distance (*p* < 0.001, robust *R*^2^*adj* = 0.22 [moderate effect size]) and the total landing developmental sequence score (*p* = 0.03, robust *R*^2^*adj* = 0.17 [moderate effect size]), but not the TGMD-3 horizontal jump (*p* = 0.09, robust *R*^2^*adj* = 0.12 [weak effect size]). As a large percentage of individuals landed stiffly, scale attenuation (i.e., ceiling) effects were a potential concern. However, as hindered performance was common across many of the SLJ assessments within the current sample, landing stiffly (i.e., impaired performance) appeared to (in general) parallel other forms of substandard SLJ performance. While this was not statistically true when the TGMD-3 horizontal jump was the response variable, the LESS displacement item *p*-value was <0.10 and the effect size was weak (as opposed to trivial). Therefore, it seems as though the LESS item leaned toward being predictive of the TGMD-3 horizontal jump in youth with VI. However, further study is needed. 

Results were also quite telling for the regression analyses whereby numerous variables of interest were individually regressed across the three primary SLJ assessment scores. Overall, our hypothesis was incorrect as very few of the investigated variables were predictive of SLJ performance in youth with VI. One of the biggest discrepancies was the lack of predictiveness of biological sex and age on SLJ distance. Specifically, boys and those who are older in age typically jump longer SLJ distances [18,19,20]. Maturity offset nor a slew of biomechanical variables (e.g., height, leg length) were predictive of the three SLJ assessments. This was surprising as maturation and growth in youth should, in theory, lead to numerous characteristics, which ought to benefit SLJ performance (e.g., increased neuromuscular capabilities/muscle mass, longer lever arms). Using existing data, our aforementioned descriptive-comparative results suggested that youth with VI may have deficient SLJ characteristics compared to youth without VI (i.e., inter-population issue). However, based on the regression results and historical, comparable trends that have been noted in peers without VI, the lack of discrimination/predictiveness amongst the investigated variables of interest highlights that youth with VI may also have intra-population SLJ deficits/concerns. 

While many potential variables of interest were not predictive of SLJ performance, being multimorbid had a statistically significant but weak predictive effect in relation to the TGMD-3 horizontal jump (*p* = 0.006; robust *R*^2^*adj* =0.09) and maximal SLJ distance (*p* = 0.001; robust *R*^2^*adj* =0.12). However, opposing results were found for the total landing developmental sequence score (*p* = 0.87; robust *R*^2^*adj* = 0.00). The lack of predictiveness for the total landing developmental sequence score across multimorbidity status was somewhat surprising. A clear explanation for this outcome remains elusive. Yet, based on this result, it appears that landing the SLJ (using a summed, qualitative score) is a skill that is not discriminative across multimorbidity status in youth with VI. It is important to remember that multimorbidity status was dichotomous (i.e., yes/no). Future research may consider investigating different or expanded multimorbidity categories and their effect on SLJ performance as such analyses may yield different (or more detailed) findings. 

Yet, the totality of the multimorbidity results (i.e., significant predictor for two out of three SLJ assessments) provide credence to the recommendation that practitioners and researchers alike should acknowledge and/or consider multimorbidity as a potential variable of influence in youth with VI [46]. Specifically, it is reasonable to suggest that if an individual with a VI has at least one additional chronic condition that said condition(s) may be responsible for a substantial amount of shared or unique variance concerning SLJ performance. This sentiment is reinforced by the conclusions of Crews and colleagues [80] who suggested that individuals with VI who are multimorbid may be at risk for ‘double jeopardy’ (i.e., decreased physical/mental health due to the compounding effects of coexisting chronic conditions). 

The only remaining statistically significant predictor was vision level, which was weakly predictive of the total landing developmental sequence score (*p* = 0.03; robust *R*^2^*adj* = 0.03). This finding suggests that qualitative SLJ landing performance (as represented by a summed score) may indeed be influenced by vision level—but only to a minor effect—which would support previous hypotheses/findings [11]. This outcome was in direct contrast to the corresponding vision level regressions. Specifically, vision level did not predict TGMD-3 horizontal jump (whose criteria partially focuses on the takeoff phase; *p* = 0.46; robust *R*^2^*adj* =0.00) or maximal SLJ jump distance (i.e., a product measure; *p* = 0.18; robust *R*^2^*adj* =0.00). This result suggests that vision level may (in particular) be a factor of consideration for landing the SLJ (from a qualitative perspective) in youth with VI. As previously discussed, this outcome is likely due to the fact that vision can assist with temporo-spatial accuracy during motor skill performance (e.g., visuo-motor integration). Interestingly, the mixed results echo the literature as vision level has been shown to [45] and not to [58] impact motor skill performance in youth with VI. The current results emphasize that vision level may be a differential predictor of SLJ performance (i.e., its predictiveness is not constant, it depends what assessment/measurement is being used).

Based on the current results, it appears that youth with VI have lower SLJ characteristics with respect to extant data in youth without VI. Not only were SLJ scores substandard (based on descriptive statistics and extrapolated developmental expectations), but numerous discrepancies were uncovered which contrasted with typical trends (e.g., age, sex were not predictive of SLJ performance). While the default assumption may be that having a VI (in and of itself) would be the primary culprit of such outcomes (i.e., VI = primary structural constraint), such results were not endorsed within the current study. In fact, while a complex interaction of personal, environmental, and social factors influence motor skill performance [58,77], it is likely that socio-environmental constraints are some of the most impactful determinants of motor skill performance [81], physical activity [82], and health [83] in youth with VI. While these data cannot corroborate that socio-environmental factors were the motive force behind the inferior SLJ scores, future research should investigate such hypotheses especially given that, for example, modifiable environmental factors have been found to influence motor skill performance in younger children without VI [84]. This is important as process- and product-oriented SLJ performance can be improved with targeted, professional-led intervention in youth with VI [81]. 

Overall, there are serious implications that can be derived from the current study. As the current sample appeared to struggle with developing and harnessing explosive strength during the SLJ, it is reasonable to suggest that youth with VI may have a compromised health status [1] and/or impoverished muscular fitness [7], which could have acute and longitudinal consequences [3,4]. It may be that youth with VI have blunted developmental trajectories of health [85]. At present, youth with VI may be experiencing a negative spiral of disengagement surrounding movement-related behaviors and outcomes due to their lack of motor skillfulness and explosive muscular strength as measured via the SLJ (i.e., lack of motor skill ↔ lack of fitness/physical activity [86]). However, the cause-and-effect directionality (i.e., reciprocity) of such hypotheses requires further investigation.

Moving forward, youth with VI should be provided with early, consistent, accessible, socio-environmental-conscious, and professional-led interventions in which they are able to develop motor skills (such as the SLJ) and myo-specific characteristics such as explosive strength. If afforded such interventions, youth with VI will likely achieve a level of motor skill and muscular fitness that will enable them to regularly and successfully participate in goal-directed, translational, and health-enhancing levels of movement [71,86]. Specifically, if youth with VI achieve a certain threshold of skill across all SLJ phases (i.e., they meet and/or surpass a ‘proficiency barrier’ for the SLJ takeoff, flight, landing), such youth could be described as having a mobility (as opposed to a stability) profile [71], which would likely have a positive, cascading effect on various life domains (e.g., activity, participation, body function/structure). 

The current study is not without limitations. For example, all participants attended a week-long residential sports camp, in one of two Eastern states within the United States, and were recruited via convenience sampling. Thus, generalizability concerns remain. Concerns could also be raised about the landing instructions or guidelines utilized during the SLJ attempts (i.e., participants were not told that they must stick the landing for the trial to count). However, not having such instructions allowed for an authentic assessment of the SLJ and enabled the use of the TGMD-3. Likewise, the current study only considered a single, discrete SLJ. Moving forward, consideration should be given toward various SLJ task constraints that are (for example) more serial in nature (e.g., SLJ to a lateral jump). Arguably, such tasks are more applied and therefore may have increased usefulness in more practical and complex scenarios (e.g., sport/game participation). 

Some may be concerned that only two trials were performed. However, taking the maximum value of two trials is a common practice in performance testing [7]. Likewise, the TGMD-3 horizontal jump is determined by summing scores across two trials [26]. Further, there were not statistically significant differences between trials one and two for the SLJ values (see Table 1 footnote) suggesting that additional trials may not have yielded different performance/variance within the current sample. It is important to note that two out of the four TMGD-3 horizontal jumping criteria were concerned with the landing phase of the horizontal jump (i.e., it is not a pure SLJ takeoff phase assessment). However, the TMGD-3 landing criteria were not as descriptive or utilitarian as the SLJ landing developmental sequences provided by Lane and colleagues [10]. 

Next, in an attempt to aid in the descriptive interpretation of SLJ performance in youth with VI, age- and sex-specific percentile rank normative values were calculated [18,19,20]; however, such norms had temporal and population-related shortcomings. Further, individual component percentages of occurrence for the landing developmental sequence assessment that were used for global comparison were extrapolated from Lane et al. [10]. Such values were based on cross-sectional data, were estimates, and were determined by using the rough average age of the sample as the referent timepoint (≈13 years). Thus, such values should only be viewed as a guiding heuristic. Finally, the variables of interest that were investigated within the regression analyses were not an exhaustive list. Future consideration should be given to additional variables including (but not limited to) socio-environmental factors. 

## 5. Conclusions

The primary charge of this study was to complete an extensive and multifaceted investigation of the SLJ, a lower-body explosive strength field test, in youth with VI—the first of its kind. Using a multiphase and hybrid orientation, our results suggested that youth with VI have relatively low process- and product-based SLJ performance when compared to previously published data in youth without VI. Such results should be viewed with earnestness as SLJ distance is a general index of muscular fitness in youth [7] and muscular fitness is an important marker of health in youth [1] that may track into adulthood [3,4]. However, these results should act as a precedent (and provide an impetus) for hybrid and multiphase SLJ investigations, as it is possible that qualitative-type assessments may provide enhanced and/or novel information (compared to SLJ distance) concerning various health- or performance-related outcomes in youth with VI. 

Additional concern should be raised due to the fact that most variables of interest were not predictive of SLJ scores. Such discrepancies (when compared to what is expected in youth without VI) should be viewed as an additional red flag. Moving forward, researchers and practitioners may want to consider the influence of the included variables as well as other factors that may be suspected of influencing SLJ performance. Multimorbidity status is a variable that may need regular consideration surrounding the SLJ in youth with VI.

## Figures and Tables

**Table 1:** Descriptive statistics for individual SLJ trials and the isolated (max) trial, which was composed of scores from the trial with the longest SLJ distance.

Variable	Trial 1	Trial 2	Max Trial ^1^
	M (SD)	M (SD)	M (SD)
Distance (m)	1.08 (0.48)	1.06 (0.52)	1.15 (0.48)
Total Dev. Seq.	5.36 (1.52)	5.25 (1.36)	5.31 (1.36)
LESS Joint Disp.	1.66 (0.63)	1.64 (0.68)	1.59 (0.72)
Suppl. Strategy	0.87 (0.90)	0.92 (0.88)	0.82 (0.90)
	**Mdn (MAD)**	**Mdn (MAD)**	**Mdn (MAD)**
Distance (m)	1.11 (0.42)	1.15 (0.49)	1.21 (0.39)
Total Dev. Seq.	5.00 (1.48)	5.00 (1.48)	5.00 (1.48)
LESS Joint Disp.	2.00 (0.00)	2.00 (0.00)	2.00 (0.00)
Suppl. Strategy	1.00 (1.48)	1.00 (1.48)	1.00 (1.48)
	**Skew; Kurt**	**Skew; Kurt**	**Skew; Kurt**
Distance (m)	−0.48; 0.02	−0.40; −0.37	−0.45; 0.06
Total Dev. Seq.	0.49; −0.41	0.77; 0.52	0.57; 0.34
LESS Joint Disp.	−1.58; 1.20	−1.58; 0.95	−1.38; 0.34
Suppl. Strategy	0.65; −0.63	0.59; −0.58	0.89; −0.10

^1^ 51% (*n* = 31) of participants jumped the furthest distance on the trial 1; 49% (*n* = 30) of participants jumped the furthest distance on trial 2. Note. Per Exact Wilcoxon signed-rank tests with the Pratt modification, no statistically significant differences (*p* > 0.05) were found between trial 1 and 2.

**Table 2:** Percentages of occurrence for individual SLJ trials and the isolated (max) trial, which was composed of scores from the trial with the longest SLJ distance.

Variable	Trial 1	Trial 2	Max Trial ^2^
	%	%	%
Common Component Profiles ^1^			
1-1-1	9.84	6.56	8.20
1-1-2	---	9.84	6.56
1-2-1	**14.75**	**13.11**	9.84
1-2-2	**36.07**	**32.79**	**37.70**
1-2-3	---	9.84	8.20
1-2-4	8.20	---	---
2-2-2	---	9.84	**11.48**
2-2-3	6.56	---	---
2-2-4	6.56	---	---
Component—Shank			
1	78.69	78.69	77.04; 20 ^† 3^
2	21.31	21.31	22.96; 80 ^† 3^
Component—Foot			
1	13.11	19.67	14.75; 15 ^† 3^
2	81.97	73.77	80.33; 55 ^† 3^
3	4.92	6.56	4.92; 30 ^† 3^
Component—Arm			
1	24.59	19.67	18.03; 15 ^† 3^
2	45.90	55.74	57.38; 45 ^† 3^
3	11.48	13.11	13.11; 15 ^† 3^
4	18.03	11.48	11.48; 25 ^† 3^
LESS Joint Disp.			
0 (Soft)	8.20	11.48	13.12
1 (Average)	18.03	13.11	14.75
2 (Stiff)	73.77	75.41	72.13
Suppl. Strategy			
0 (None)	42.62	37.70	44.26
1 (Mild)	32.79	37.70	36.07
2 (Moderate)	19.67	19.67	13.11
3 (Severe)	4.92	4.92	6.56

^1^ Top six profiles per trial (i.e., not an exhaustive list of all possible combinations/profiles); two most common profiles per trial have been bolded. ^2^ 51% (*n* = 31) of participants jumped the furthest distance on the trial 1; 49% (*n* = 30) of participants jumped the furthest distance on trial 2. ^3^ Dagger (^†^) values are extracted and estimated percentages of occurrence from Lane et al. [10] for a 13-year-old (i.e., the rough average age of the current sample).

**Table 3:** Individual robust bivariate regression results between SLJ measures.

Predictor (*x*)	*β*	*SE*	*t*-Value	*p*	Robust *R*^2^*adj*
	**TGMD-3 Horiz. Jump (*y*)**				
*β_0_*	1.28	0.71	1.81	0.08	
***β_1_*: Max. SLJ Distance (m)**	**2.95**	**0.57**	**5.16**	<0.001 ***	**0.37**
*β_0_*	−0.81	1.01	−0.80	0.43	
***β_1_*: Total Dev. Seq.**	**1.00**	**0.19**	**5.41**	<0.001 ***	**0.32**
*β_0_*	6.54	1.27	5.14	<0.001 ***	
*β_1_*: LESS Joint Disp.	−1.28	0.74	−1.73	0.09	0.12
*β_0_*	4.43	0.57	7.74	<0.001 ***	
*β_1_*: Suppl. Strategy	0.21	0.46	0.45	0.66	0.00
	**Max. SLJ Distance (*y*)**				
*β_0_*	0.57	0.17	3.48	<0.001 ***	
***β_1_*: TGMD-3 Horiz. Jump**	**0.13**	**0.03**	**3.94**	<0.001 ***	**0.31**
*β_0_*	−0.14	0.25	−0.56	0.58	
***β_1_*: Total Dev. Seq.**	**0.24**	**0.05**	**5.25**	<0.001 ***	**0.38**
*β_0_*	1.67	0.14	11.80	<0.001 ***	
***β_1_*: LESS Joint Disp.**	**−0.30**	**0.08**	**−3.66**	<0.001 ***	**0.22**
*β_0_*	1.21	0.09	13.18	<0.001 ***	
*β_1_*: Suppl. Strategy	0.01	0.08	0.13	0.90	0.00
	**Total Dev. Seq. (y)**				
*β_0_*	3.81	0.28	13.43	<0.001 ***	
***β_1_*: TGMD-3 Horiz. Jump**	**0.30**	**0.06**	**5.18**	<0.001 ***	**0.33**
*β_0_*	3.35	0.41	8.11	<0.001 ***	
***β_1_*: Max. SLJ Distance (m)**	**1.67**	**0.34**	**4.89**	<0.001 ***	**0.42**
*β_0_*	6.55	0.65	10.15	<0.001 ***	
***β_1_*: LESS Joint Disp.**	**−0.84**	**0.37**	**−2.27**	0.03 *	**0.17**
*β_0_*	4.94	0.29	16.98	<0.001 ***	
*β_1_*: Suppl. Strategy	0.22	0.24	0.92	0.36	0.00

Note. * and *** denote ≤0.05 and ≤0.001, respectively. Statistically significant predictors have been bolded.

**Table 4:** Individual robust bivariate regression results for the potential variables of influence.

Predictor (*x*)	*β*	*SE*	*t*-Value	*p*	Robust *R*^2^*adj*
	**TGMD-3 Horiz. Jump (*y*)**				
*β_0_*	5.06	3.17	1.59	0.12	
*β_1_*: Age	−0.04	0.24	−0.15	0.88	0.00
*β_0_*	4.29	0.84	5.12	<0.001 ***	
*β_1_*: Sex	0.50	1.14	0.43	0.67	0.00
*β_0_*	4.56	0.56	8.08	<0.001 ***	
*β_1_*: Maturity Offset	−0.10	0.31	−0.31	0.76	0.00
*β_0_*	5.95	6.02	0.99	0.33	
*β_1_*: Height	−0.88	3.89	−0.23	0.82	0.00
*β_0_*	4.97	6.15	0.81	0.42	
*β_1_*: Limb Length	−0.46	7.29	−0.06	0.95	0.00
*β_0_*	4.79	1.57	3.05	0.003 **	
*β_1_*: Weight	−0.01	0.03	−0.16	0.88	0.00
*β_0_*	4.90	0.68	7.25	<0.001 ***	
*β_1_*: BMIz	−0.37	0.53	−0.69	0.49	0.00
*β_0_*	3.73	1.24	3.02	0.004 **	
*β_1_*: Vision Level	0.37	0.50	0.74	0.46	0.00
*β_0_*	5.34	0.37	14.60	<0.001 ***	
***β_1_*: Multimorbidity**	**−1.81**	**0.64**	**−2.84**	0.006 **	**0.09**
	**Max. SLJ Distance (*y*)**				
*β_0_*	1.07	0.44	2.42	0.02 ***	
*β_1_*: Age	0.01	0.03	0.32	0.75	0.00
*β_0_*	1.10	0.12	9.12	<0.001 ***	
*β_1_*: Sex	0.18	0.16	1.14	0.26	0.01
*β_0_*	1.21	0.07	16.80	<0.001 ***	
*β_1_*: Maturity Offset	−0.02	0.04	−0.38	0.70	0.00
*β_0_*	1.01	0.86	1.18	0.24	
*β_1_*: Height	0.13	0.56	0.24	0.81	0.00
*β_0_*	0.88	0.86	1.03	0.31	
*β_1_*: Limb Length	0.40	1.01	0.39	0.70	0.00
*β_0_*	1.42	0.19	7.51	<0.001 ***	
*β_1_*: Weight	−0.004	0.003	−1.12	0.27	0.00
*β_0_*	1.29	0.08	14.43	<0.001 ***	
*β_1_*: BMIz	−0.11	0.07	−1.60	0.12	0.04
*β_0_*	1.01	0.15	6.56	<0.001 ***	
*β_1_*: Vision Level	0.08	0.06	1.37	0.18	0.00
*β_0_*	1.33	0.06	21.07	<0.001 ***	
***β_1_*: Multimorbidity**	**−0.38**	**0.11**	**−3.43**	0.001 **	**0.12**
	**Total Dev. Seq. (*y*)**				
*β_0_*	4.10	1.06	3.87	<0.001 ***	
*β_1_*: Age	0.08	0.08	1.02	0.31	0.00
*β_0_*	4.90	0.25	19.80	<0.001 ***	
*β_1_*: Sex	0.56	0.35	1.60	0.11	0.03
*β_0_*	5.16	0.17	29.72	<0.001 ***	
*β_1_*: Maturity Offset	0.04	0.10	0.42	0.68	0.00
*β_0_*	2.42	2.38	1.02	0.31	
*β_1_*: Height	1.79	1.54	1.16	0.25	0.00
*β_0_*	3.72	2.18	1.71	0.09	
*β_1_*: Limb Length	1.72	2.59	0.66	0.51	0.00
*β_0_*	4.73	0.48	9.76	<0.001 ***	
*β_1_*: Weight	0.01	0.01	0.95	0.35	0.00
*β_0_*	5.24	0.21	25.15	<0.001 ***	
*β_1_*: BMIz	−0.15	0.17	−0.90	0.38	0.00
*β_0_*	4.37	0.40	11.19	<0.001 ***	
***β_1_*: Vision Level**	**0.35**	**0.16**	**2.22**	0.03 *	**0.03**
*β_0_*	5.17	0.21	25.06	<0.001 ***	
*β_1_*: Multimorbidity	−0.07	0.39	−0.17	0.87	0.00

Note. *, **, and *** denote ≤0.05, ≤0.01, and ≤0.001, respectively. Statistically significant predictors have been bolded.

## Data Availability

The data presented in this study are available on request from the corresponding author.
